# Supplementary material for: Mapping cellular-scale internal mechanics in 3D tissues with thermally responsive hydrogel probes
Source: Nat Commun. 2020 Sep 21;11:4757. doi: 10.1038/s41467-020-18469-7 (PMC7505969; doi:10.1038/s41467-020-18469-7)
Supplement: Supplementary file 1 — Supplementary Information [file 41467_2020_18469_MOESM1_ESM.pdf]

Supplementary Information

Mapping cellular-scale internal mechanics in 3D tissues with thermally responsive hydrogel probes

Stephanie Mok[1], Sara Al Habyan[2], Charles Ledoux[1], Wontae Lee[1], Katherine MacDonald[1], Luke McCaffrey[2] and Christopher Moraes[1,2,3,*]

Affiliations
[1] Dept. of Chemical Engineering; McGill University, Montreal, QC, Canada
[2] Goodman Cancer Research Centre; McGill University, Montreal, QC, Canada
[3] Dept. of Biomedical Engineering, McGill University, Montreal, QC, Canada

Supplementary Figures

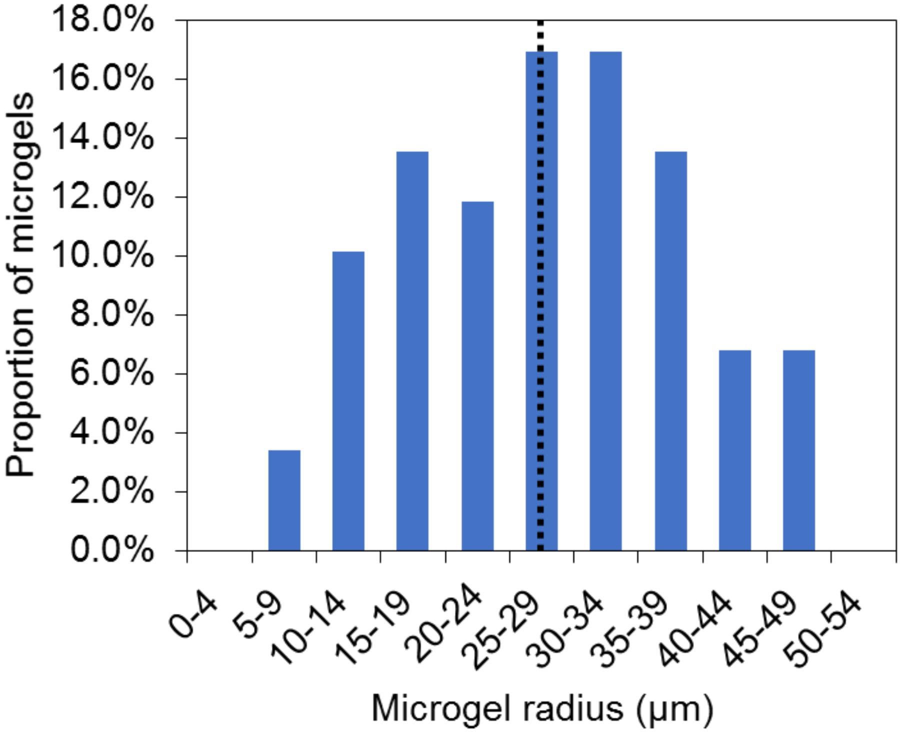

Supplementary Figure 1. NiPAAM microgel size distribution at room temperature. Polydispersed microgels fabricated using the oil/water emulsion technique show a normal distribution of sizes with a mean radius (dotted line) of 27±11 µm. Data reported for µTAMS in expanded state (n=59).

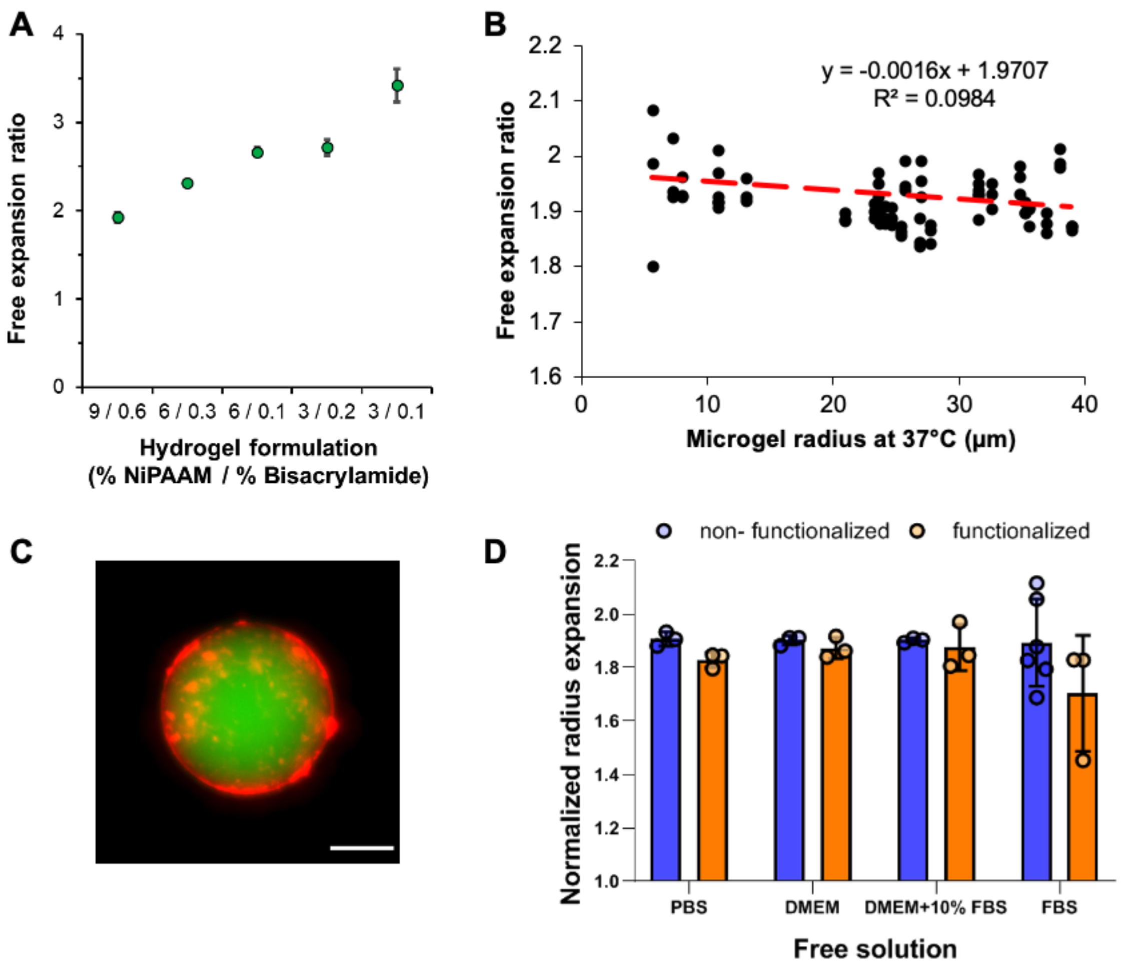

Supplementary Figure 2. Unconfined thermoresponsive free expansion of μTAMs in solution. (A) The free expansion ratio between the measured diameters of microgels in their expanded and contracted state varies for various tested hydrogel formulations (n= 10, 11, 11, 8 and 9 individual μTAMs in order shown on graph). Data points show mean +/- SD. (B) Free expansion ratios are independent of microgel size (data presented for PNiPAAM formulation 9N0.6B (see Suppl. Table S1). Red dashed line indicates linear trend where y= 0.-0016x +1.9707, R^2 = 0.0984; n = 26). No correlation was observed between PNiPAAM microgel size and expansion ratio. Increased variability for smaller microgel sizes can be attributed to increased measurement error percentage under the selected imaging conditions . (C) Representative image of a μTAM (green) functionalized with type I collagen (red). Scale bar = 25 μm. (D) Comparison of free expansion ratios for native and surface functionalized microgels in solutions with varying fetal bovine serum (FBS) protein content. Functionalization with collagen I has no statistically significant effect in PBS, DMEM, complete media (DMEM + 10%FBS), or 100% FBS, but some increased variability is observed in protein-rich suspensions. (Data presented as mean ± SD, statistical analysis conducted by two-way ANOVA, not significant p = 0.56, 0.47 and 0.09 for the interaction between solutions and surface coating, within solutions, and within coatings; n = 3 for PBS, DMEM, DMEM+10% FBS, and functionalized gels in FBS; n= 6 for non-functionalized gels in FBS). Representative images from three collagen-I staining experiments from two separate μTAM samples.

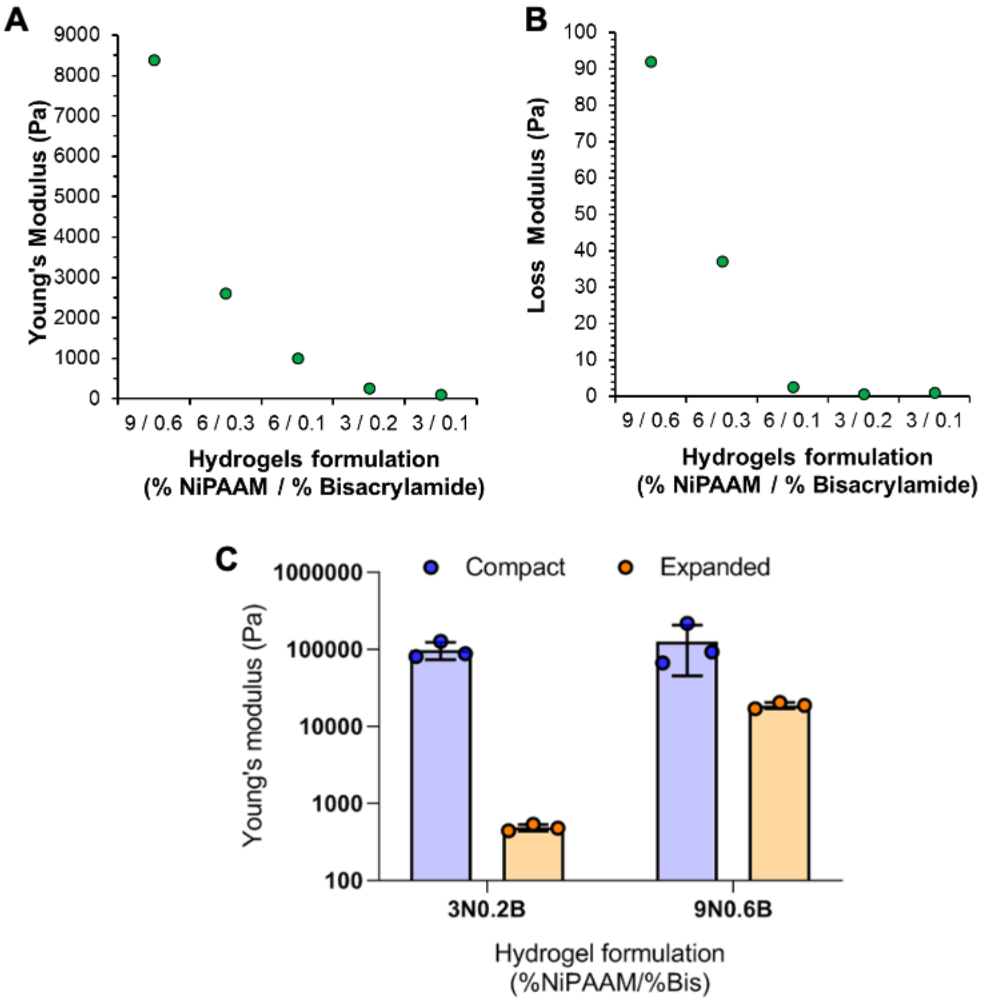

Supplementary Figure 3. Mechanical characteristics of PNiPAAM obtained via shear rheology. (A) PNiPAAM hydrogel storage modulus and (B) loss modulus. Loss moduli were minimal, indicating strongly linear elastic materials behavior. (C) PNiPAAM stiffness at the compact (>34 °C) and expanded (<34 °C) hydrogel state indicate that stiffness changes dramatically between the compact and expanded state depending on PNiPAAM formulation. Data reported as mean ± SD for n = 3.

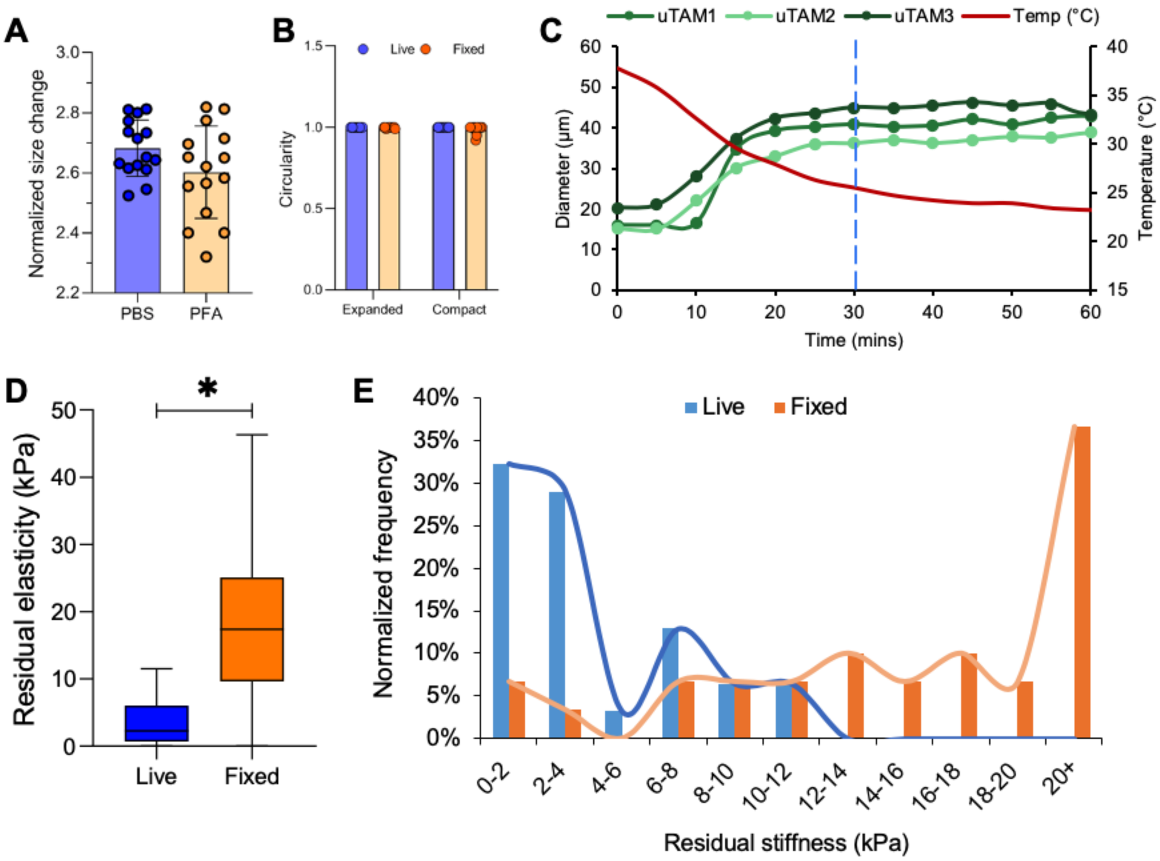

Supplementary Figure 4. Residual elasticity distribution within engineered T47D multicellular aggregates before and after fixation. T-47D mammary-derived ductal carcinoma cells formed spheroids over 2 days within polyacrylamide micropockets and fixed with 4% paraformaldehyde overnight at 37°C. (A) Fixation does not affect free µTAM swelling characteristics. (n = 15 µTAMs readings for each condition). (B) Circularity of µTAMs within live and fixed tissues. No significant differences ($p = 0.061$) in circularity between compact and expanded µTAMs in either tissue condition are seen based on a two-way ANOVA. Data reported for n = 16 and 17 for live and fixed tissue condition respectively in each µTAM state. (C) µTAM size change over time within T47D spheroids during cooling on our microscope stage. Temperature and size measurements were recorded every 5 minutes for an hour to ensure that 30 minutes was adequate time for µTAM expansion to equilibrate, thereby ensuring residual elasticity around the µTAM is measured. (D) Stiffness in fixed spheroids is significantly stiffer than its live pre-fixed state (n = 33 and 30 individual µTAM readings in live and fixed spheroids respectively; 30 live spheroids and 11 fixed spheroids were measured). Asterisks denotes $p < 0.0001$ according to unpaired two-tailed t-test with Welch's correction. (E) Histogram of residual elasticity distributions between live and fixed spheroids. Data from panels A, B and D are presented as mean +/- SD.

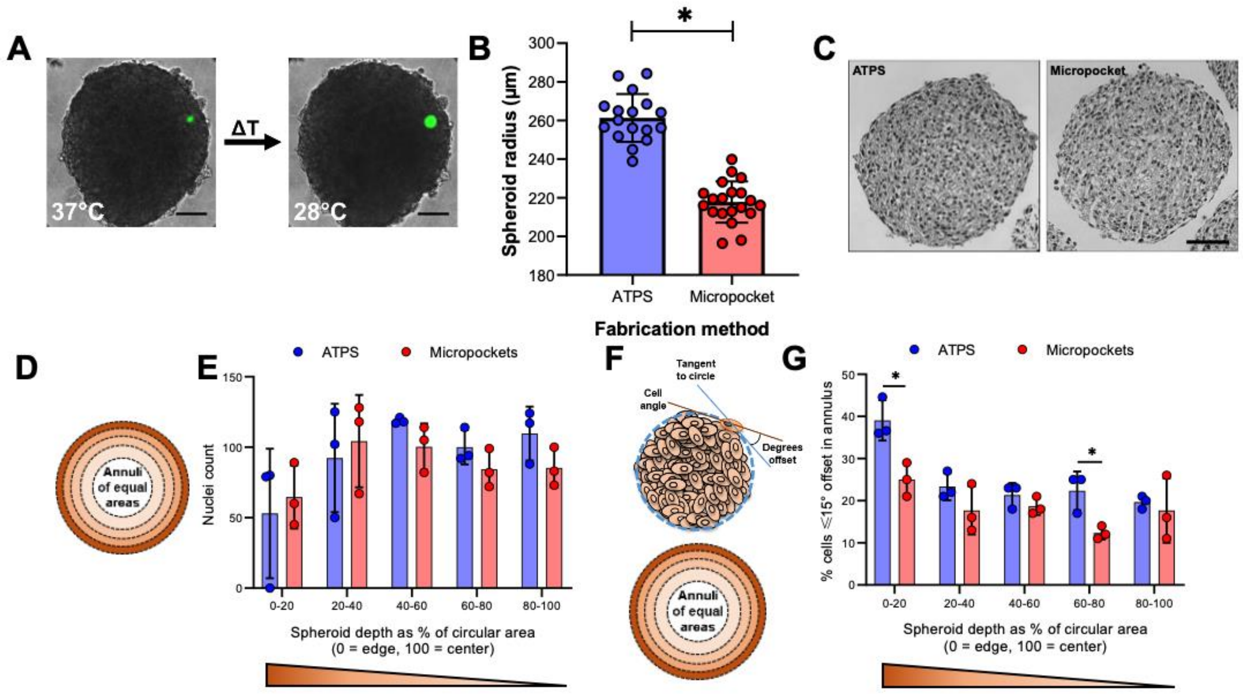

Supplementary Figure 5. Cell count and orientation in ATPS and micropocket spheroids. (A) Spheroids are formed and maintained at 37 °C, where µTAMs remain compacted. Measurements of residual stiffness can be made by reducing the temperature and expanding the µTAMs. Scale bar = 100 µm. (B) Characterization of spheroid dimensions formed in ATPS and micropocket systems. ATPS spheroids are slightly but significantly larger in radius than micropocket spheroids by 44 ± 4 µm (unpaired two-tailed t-test, $p < 0.0001$ for n = 20 and 17 respectively). (C) Representative images of H&E stained sections show that at the edge, ATPS spheroids preferentially align along the circumference of the spheroid in contrast to micropocket spheroids. Scale bar = 100 µm. (D, E) Nuclei count between the two spheroid generation methods in each annuli representing equal areas show no significant differences. (n = 3). (F, G) Cell orientation analysis show that cells along the periphery of ATPS spheroids preferentially elongate along the circumference of the spheroid significantly more than their micropocket counterparts. Asterisks denotes significance at $p = 0.0022$ by two-ANOVA with a Bonferroni test. (n = 3 spheroids for both ATPS and micropocket methods). Representative images show one example out of hundreds of spheroids generated with µTAMs incorporated over at least 6 independent HS-5 spheroid generation experiments for each method. Data for panels B, E and G are presented as mean +/- SD.

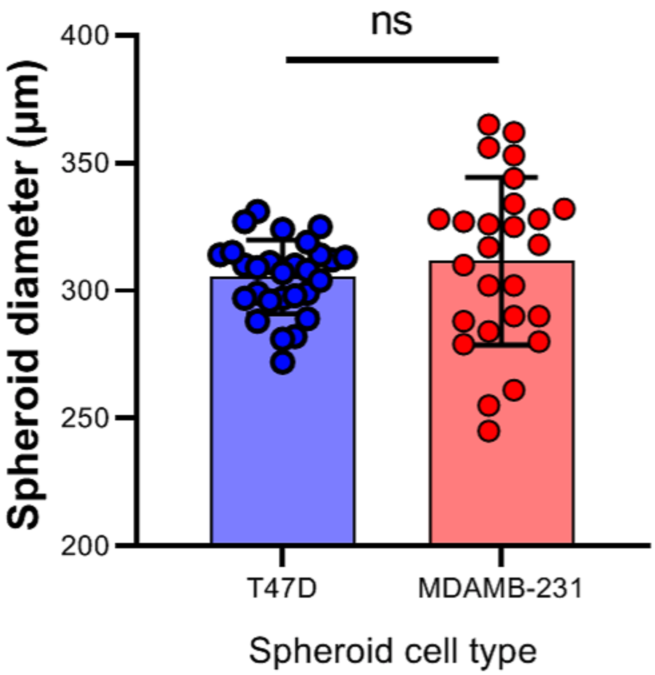

Supplementary Figure 6. Diameter of breast cancer cell line spheroids generated using polyacrylamide micropockets. No significant difference in spheroid size between the two cell types (unpaired two-tailed t-test with Welch's correction; n.s. $p = 0.383$, $n = 28$ and 26 for T47D and MDA-MB-231 spheroids respectively). Data are presented as mean values +/- SD.

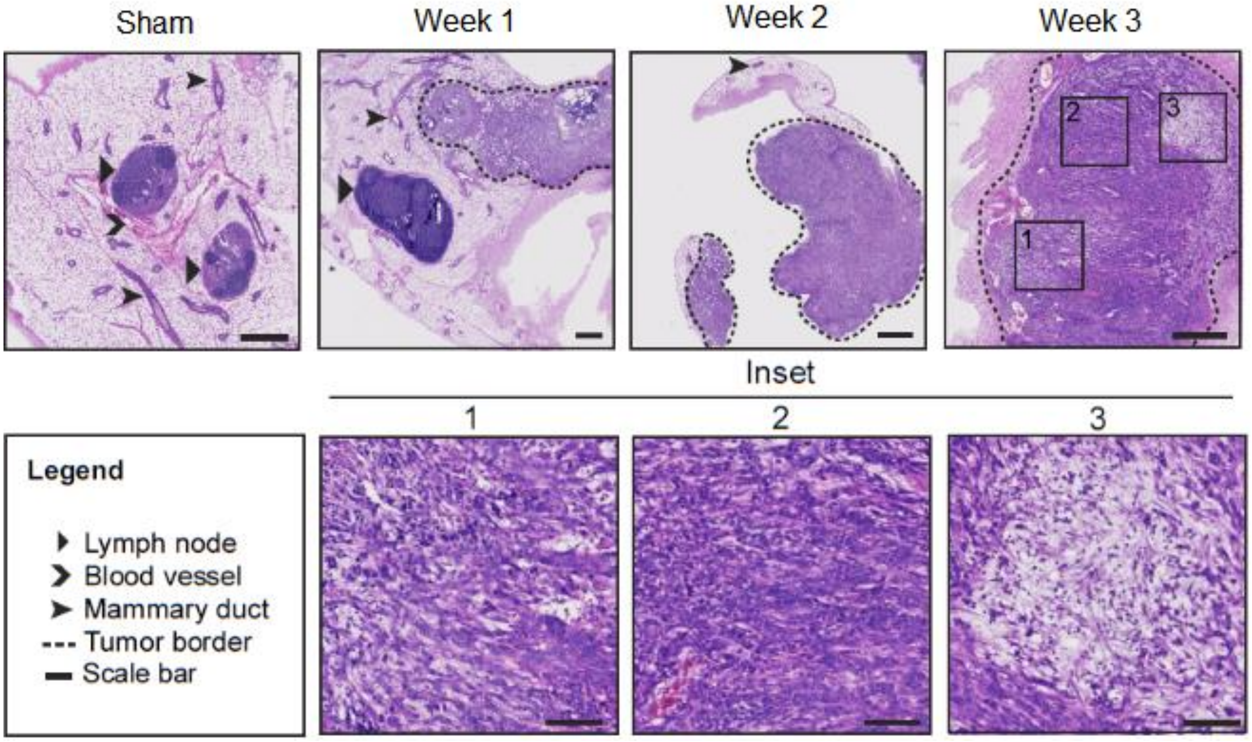

Supplementary Figure 7. Tumour development over three weeks. Representative images of T41 tumour growth within the mammary fat pad of immunocompetent BALB/c mice. Scale bars = 500 µm. Inset scale bars 100 µm. Representative images showing consistent sightings among 6 BALB/c mice injected with 4T1 cancer cells + µTAMs in both left and right mammary ducts and 6 sham mice injected with PBS + µTAMs.

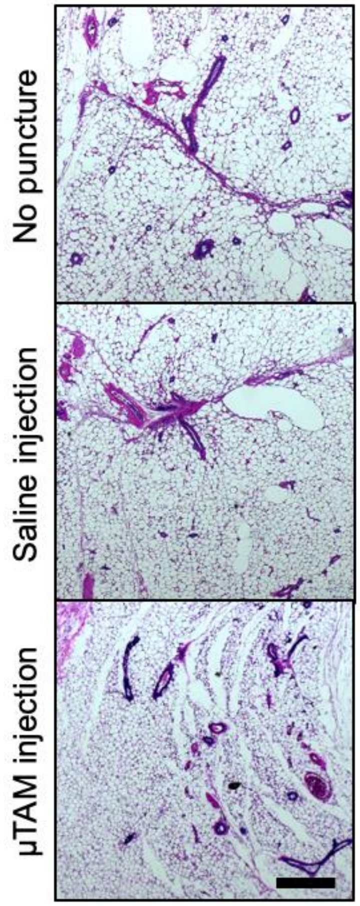

Supplementary Figure 8. Comparative histology in mouse mammary fat pads subjected to needle injection. Representative images of mammary fat pads that were injected with saline or µTAMs using a 22G needle compared to fat pads that were not punctured show no visual differences in histology with an H&E stain. Seen in these sections are normal fat cells with occasional cross and longitudinal sections of mammary duct structures and blood vessels. Fibrotic tissue would appear as dense areas of collagen which would stain pink. Scale bar = 500 µm. Two mice were tested in both left and right mammary ducts for n = 4 for each condition.

Supplementary Tables

Supplementary Table 1. PNiPAAM microgel formulations. Highlighted row in yellow indicates formulation used for all in vitro and in vivo experiments.

NiPAAM (%)	Bis-acrylamide (%)	Formula shorthand	20% (w/v) NiPAAM in PBS (µL)	2% Bis-acrylmide (µL)	PBS (µL)	TEMED (µL)	Fluorescein o-methacrylate in DMSO (100 mg/mL)	1% (w/v) APS in PBS (µL)
9	0.6	9N/0.6B	450	300	147.5	1.5	1	100
6	0.3	6N/0.3B	300	150	447.5	1.5	1	100
6	0.1	6N/0.1B	300	50	547.5	1.5	1	100
3	0.2	3N/0.2B	150	100	647.5	1.5	1	100
3	0.1	3N/0.1B	150	50	697.5	1.5	1	100

Supplementary Table 2. Tissue phantom formulations of stiffness-tunable polyacrylamide.

Acrylamide (%)	Bis-acrylamide (%)	40 % Acrylamide (µL)	2% Bis-acrylmide (µL)	PBS (µL)	TEMED (µL)	1 or 10% (w/v) APS in PBS (µL)	Young's Modulus based on shear rheology (Pa)
3	0.05	75	24.5	799	1.5	100 (1%)	150
3	0.11	75	53.5	770	1.5	100 (1%)	400
7.5	0.05	187.5	27	694	1.5	100 (1%)	4250
7.5	0.24	187.5	118	593	1.5	100 (1%)	9200
12	0.24	300	120.5	478	1.5	100 (1%)	19500
20	2	490	300	304.5	0.5	5 (10%)	245000
20	3	485	200	204.5	0.5	5 (10%)	271000

Supplementary Table 3. Best fit values for PNiPAAM expansion model (based on Equation (2)). Asterisks show empirically measured values. Bolded values are iterated best fits

Polyacrylamide Formulation	$E_{microgel}$ (kPa) Expanded - Contracted	Free expansion ratio	B coefficient	R^2 fit
**3N0.2B**	0.48 * - 98 *	2.72*	N/A	N/A
	**12.45**	**2.52**	**0.91**	**0.94**
**9N0.6B**	18.6* - 125*	1.92*	0.34	0.86
	**21.60**	**1.78**	**0.514**	**0.91**

Supplementary Table 4. Residual elasticity mapping data for HS-5 spheroids.

Fabrication method	Spheroid radius (µm)	µTAM distance from edge (µm)	µTAM radius (um)		Ratio (expanded /compact)	Residual stiffness (Pa)
			Compact	Expanded		
ATPS	239	45	8.9	17.8	2.00	5968
	252	40	9.6	18.9	1.96	6838
	283	103	8.4	20.4	2.43	610
	283	81	5.8	11.6	2.00	6002
	265	39	10.4	18.5	1.77	11915
	284	37	6.9	14.3	2.08	4545
	284	50	7.4	16.4	2.22	2662
	256	53	15.3	30.5	1.99	6303
	260	58	10.5	23.1	2.20	2825
	255	42	9.7	18.5	1.91	8030
	255	110	5.6	12.1	2.15	3536
	264	31	6.7	16.4	2.45	457
	250	55	4.0	7.3	1.81	10882
	276	50	14.0	28.6	2.04	5321
	257	47	7.0	14.7	2.10	4270
	267	44	12.2	30.2	2.48	236
	268	69	7.4	15.1	2.06	4989
	268	71	5.4	11.1	2.06	4935
	264	82	14.6	25.9	1.78	11834
	281	21	8	15	1.88	8859
	281	100	19	38	2.00	6019
	281	51	12	24	2.00	6019
	281	33	16	32	2.00	6019
	281	98	37	65	1.76	12533
	281	85	22	37	1.68	15601
	270	21	11	24	2.18	3104
	267	58	15	24	1.60	19920
	309	115	23	41	1.78	11624
	309	89	12	20	1.67	16313
	309	27	15	29	1.93	7426
	237	63	13	24	1.85	9650
	237	107	13	23	1.77	12086
	223	16	18	34	1.89	8498
	250	17	7	13	1.86	9341
	250	59	15	29	1.93	7426
	250	27	19	31	1.63	18108
	258	111	31	50	1.61	19155

	258	29	15	29	1.93	7426
	258	29	14	29	2.07	4734
	258	89	11	22	2.00	6019
	234	23	19	35	1.84	9765
	281	111	13	24	1.85	9650
	281	72	11	23	2.09	4418
	234	91	21	33	1.57	21747
	234	49	14	22	1.57	21747
	234	37	22	37	1.68	15601
	242	93	14	26	1.86	9341
	242	36	10	19	1.90	8219
Micropocket	212	87	8.4	20.5	2.43	541
	212	106	11.8	24.6	2.08	4517
	216	80	8.2	18.4	2.25	2241
	216	96	8.2	17.1	2.07	4731
	230	54	6.9	15.0	2.18	3127
	223	22	5.8	13.1	2.26	2141
	223	105	8.1	17.8	2.21	2763
	215	78	4.1	7.9	1.91	7854
	215	51	4.7	9.9	2.11	4156
	222	48	7.6	17.0	2.23	2525
	213	100	7.8	17.0	2.18	3099
	198	81	11.8	26.4	2.23	2547
	213	63	7.0	13.1	1.86	9389
	213	72	13.1	23.6	1.80	11104
	219	72	10.7	21.8	2.03	5466
	219	75	9.9	20.8	2.11	4160
	240	74	13.9	32.0	2.30	1696
	228	44	7.4	16.5	2.25	2307
	228	112	7.5	13.1	1.75	12788
	196	24	7.1	14.3	2.01	5851
	196	69	9.2	17.4	1.90	8289
	212	77	6.8	16.3	2.38	944
	220	71	12.8	28.4	2.22	2671
	220	82	9.8	19.8	2.02	5561
	220	60	10.1	25.4	2.51	49
	233	68	10.6	23.3	2.20	2864
	233	95	13.6	31.6	2.32	1524
	219	91	9.7	22.8	2.37	1105
	219	97	11.8	26.6	2.27	2097
	239	45	8.9	17.8	2.00	5968
	252	40	9.6	18.9	1.96	6838
	283	103	8.4	20.4	2.43	610

283	81	5.8	11.6	2.00	6002
265	39	10.4	18.5	1.77	11915
284	37	6.9	14.3	2.08	4545
284	50	7.4	16.4	2.22	2662
256	53	15.3	30.5	1.99	6303
260	58	10.5	23.1	2.20	2825
255	42	9.7	18.5	1.91	8030
255	110	5.6	12.1	2.15	3536
264	31	6.7	16.4	2.45	457
250	55	4.0	7.3	1.81	10882
276	50	14.0	28.6	2.04	5321
257	47	7.0	14.7	2.10	4270
267	44	12.2	30.2	2.48	236
268	69	7.4	15.1	2.06	4989
268	71	5.4	11.1	2.06	4935
264	82	14.6	25.9	1.78	11834
242.5	107	24.6	43.5	1.77	12119
215.5	22	24	44.8	1.87	9081
215.5	47	24	54	2.25	2271
227	55	21	39	1.86	9341
230	36	21	40	1.90	8102
230	48	18	33	1.83	10021
237.5	29	21	42	2.00	6019
237.5	54	13	36	2.77	0

Supplementary Table 5. Residual elasticity mapping data for breast cancer spheroids.

Cell Type	Spheroid radius (µm)	µTAM distance from edge (µm)	µTAM radius (um)		Ratio (expanded /compact)	Residual stiffness (Pa)
			Compact	Expanded		
MDA-MB-231	140	29	23.4	57.0	2.44	527
	131	93	18.7	51.1	2.73	0
	128	31	22.2	54.9	2.47	264
	128	82	25.7	66.3	2.58	0
	164	99	20.6	22.9	1.11	204612
	144	49	22.7	56.3	2.48	217
	163	75	28.8	71.8	2.49	135
	145	55	23.5	53.2	2.26	2116
	142	70	27.1	66.0	2.44	531
	155	51	19.3	43.5	2.26	2163
	164	102	19.1	20.9	1.09	248360
	177	11	19.3	23.8	1.24	80910
	164	35	23.4	58.1	2.48	199
	183	94	18.9	37.3	1.97	6551
	181	54	20.1	47.1	2.34	1307
	167	116	23.7	28.1	1.19	109892
	163	90	18.4	20.2	1.10	239363
	178	40	20.7	23.8	1.15	143493
	178	94	22.9	51.2	2.24	2435
	140	34	19.9	51.7	2.60	0
	151	78	16.5	29.9	1.81	10664
	151	67	20.2	39.6	1.96	6828
	172	127	27.3	66.0	2.42	668
	123	103	16.8	19.4	1.15	137816
	166	112	21.2	49.9	2.35	1210
	145	55	14.3	31.9	2.23	2494
	159	111	23.8	59.4	2.50	119
	159	96	12.2	13.2	1.08	294621
T47D	155	76	20.1	44.0	2.19	3010
	155	122	27.9	54.0	1.94	7377
	149	119	25.0	59.6	2.38	943
	141	62	14.5	32.9	2.27	2060
	150	80	16.8	38.8	2.31	1634
	150	93	15.0	30.4	2.03	5515
	144	61	19.3	43.0	2.23	2527

144	50	10.9	20.7	1.90	8242
162	80	23.5	52.2	2.22	2608
150	84	14.3	29.4	2.06	4995
156	97	24.3	54.7	2.25	2259
156	96	14.0	27.1	1.94	7372
163	100	32.0	72.6	2.27	2062
157	51	21.7	52.1	2.40	802
149	70	18.6	40.2	2.16	3379
149	128	23.4	42.7	1.82	10276
156	88	17.0	41.7	2.45	402
157	70	17.9	39.6	2.21	2717
157	127	17.5	39.4	2.25	2255
154	97	30.7	71.6	2.33	1412
149	77	20.1	45.7	2.27	2009
166	65	15.8	33.5	2.12	3965
157	56	22.7	52.7	2.32	1515
154	83	21.7	45.8	2.11	4111
158	72	26.3	58.0	2.21	2803
160	73	22.4	49.6	2.21	2693
152	77	22.2	49.0	2.21	2780
164	100	23.7	49.3	2.08	4590
141	33	15.8	33.4	2.11	4061
155	87	25.3	57.8	2.28	1892
148	87	28.3	62.2	2.20	2897
145	75	13.0	28.4	2.18	3067
136	100	18.9	40.6	2.15	3561

Supplementary Table 6. Residual elasticity measurements from excised tumours.

Days post-injection	µTAM radius (um)		ratio (expanded /compact)	Residual stiffness (Pa)
	Compact	Expanded		
7	14.7	25.8	1.75	12632
	13.7	22.8	1.67	16276
	13.2	19.9	1.51	26856
	9.6	15.6	1.63	18121
	9.3	14.8	1.59	20623
	7.8	13.6	1.74	13219
14	7.2	14.6	2.03	5390
	8.3	14.4	1.73	13560
	8.3	13.7	1.64	17500
	8.3	11.0	1.33	51370
	9.0	16.3	1.81	10693
	12.7	22.7	1.79	11323
	11.4	19.1	1.68	15835
	14.2	28.2	1.99	6201
	10.9	19.4	1.78	11620
	11.6	23.0	1.99	6305
	10.3	20.3	1.98	6427
	15.8	28.2	1.79	11399
	18.8	39.7	2.11	4102
	8.7	16.6	1.91	7964
	16.3	31.8	1.95	7096
	9.5	17.5	1.84	9853
	10.1	15.9	1.58	21180
	14.4	23.0	1.59	20300
	7.4	10.3	1.39	40652
	11.6	19.9	1.72	13952
21	9.8	18.0	1.83	10007
	8.9	16.3	1.84	9887
	9.8	21.9	2.23	2479
	9.1	20.4	2.24	2340
	13.2	29.1	2.21	2749
	7.5	16.0	2.15	3484
	9.0	15.8	1.76	12359
	11.8	20.0	1.70	14707
	12.7	26.7	2.09	4368
	17.5	34.4	1.97	6596
	14.3	30.1	2.11	4165
	7.8	14.1	1.81	10744

	8.0	16.0	1.99	6304
	12.0	18.6	1.54	23651
	16.5	32.3	1.96	6812
	14.1	27.7	1.96	6815
	7.2	14.0	1.95	6963
	11.5	22.1	1.93	7531
	14.0	24.6	1.75	12605
	11.4	23.3	2.04	5350
	8.6	16.0	1.86	9385
	11.5	21.0	1.82	10458
	8.3	17.2	2.06	4958
	10.9	24.5	2.25	2245
	7.9	13.6	1.72	13786
	12.3	21.4	1.74	13271
	5.4	8.6	1.58	21045
	22.4	29.3	1.31	56154
	14.2	17.5	1.23	83094
	24.8	32.7	1.32	53254
	11.3	19.2	1.70	14683
	10.9	18.0	1.65	17081
	10.3	17.4	1.69	15250
28 (Sham)	10.8	20.5	1.90	8273
	7.4	10.4	1.41	37274
	7.1	10.3	1.46	30746
	12.9	19.7	1.53	24584
	6.6	12.4	1.88	8841
	6.3	10.0	1.58	21148
	10.0	20.4	2.05	5164
	9.8	17.2	1.75	12615
	5.7	11.0	1.92	7715
	8.1	15.7	1.93	7575
	12.2	21.1	1.73	13667
	11.6	16.6	1.42	35474
	13.4	20.2	1.51	26629
	8.7	12.8	1.47	30096
	9.9	19.2	1.94	7209
	7.8	15.6	2.00	5994
	8.6	14.5	1.68	15825
	11.1	19.5	1.75	12766
	10.1	20.1	1.99	6296
	11.5	21.5	1.87	8888
	6.4	13.9	2.17	3209

11.4	20.1	1.76	12456
13.9	21.2	1.52	25336
11.8	22.6	1.91	7991
5.1	10.6	2.06	4918
5.1	10.7	2.09	4438
13.0	21.8	1.69	15443
8.5	13.9	1.63	18133
