# Supplementary material for: Mapping cellular-scale internal mechanics in 3D tissues with thermally responsive hydrogel probes
Source: Nat Commun. 2020 Sep 21;11:4757. doi: 10.1038/s41467-020-18469-7 (PMC7505969; doi:10.1038/s41467-020-18469-7)
Supplement: Supplementary file 3 — Reporting Summary [file 41467_2020_18469_MOESM3_ESM.pdf]

nature research

Corresponding author(s): Christopher Moraes

Last updated by author(s): Aug 14, 2020

Reporting Summary

Nature Research wishes to improve the reproducibility of the work that we publish. This form provides structure for consistency and transparency in reporting. For further information on Nature Research policies, see our Editorial Policies and the Editorial Policy Checklist.

Statistics

For all statistical analyses, confirm that the following items are present in the figure legend, table legend, main text, or Methods section.

n/a	Confirmed	
☐	☒	The exact sample size (*n*) for each experimental group/condition, given as a discrete number and unit of measurement
☐	☒	A statement on whether measurements were taken from distinct samples or whether the same sample was measured repeatedly
☐	☒	The statistical test(s) used AND whether they are one- or two-sided<br>*Only common tests should be described solely by name; describe more complex techniques in the Methods section.*
☒	☐	A description of all covariates tested
☐	☒	A description of any assumptions or corrections, such as tests of normality and adjustment for multiple comparisons
☐	☒	A full description of the statistical parameters including central tendency (e.g. means) or other basic estimates (e.g. regression coefficient) AND variation (e.g. standard deviation) or associated estimates of uncertainty (e.g. confidence intervals)
☐	☒	For null hypothesis testing, the test statistic (e.g. *F*, *t*, *r*) with confidence intervals, effect sizes, degrees of freedom and *P* value noted<br>*Give P values as exact values whenever suitable.*
☒	☐	For Bayesian analysis, information on the choice of priors and Markov chain Monte Carlo settings
☒	☐	For hierarchical and complex designs, identification of the appropriate level for tests and full reporting of outcomes
☒	☐	Estimates of effect sizes (e.g. Cohen's *d*, Pearson's *r*), indicating how they were calculated

Our web collection on statistics for biologists contains articles on many of the points above.

Software and code

Policy information about availability of computer code

Data collection	Epi-fluorescent images collecting using Metamorph software (Olympus, Version 7.8.13.0). Live mouse tissue explants were imaged using ZEN 2012 SP5 FP3 software (Zeiss, Version 14.0.19.201). No custom code was used in this data collection
Data analysis	Images were deconvolved using the iterative deconvolve 3D plugin in FIJI (NIH, Version 1.53c). All measurements were made in FIJI. Statistical analyses performed in GraphPad Prism v8.0.2. No custom code was used in this data analysis. µTAM expansion modelling was done using FEBio v2.5.1.0.

For manuscripts utilizing custom algorithms or software that are central to the research but not yet described in published literature, software must be made available to editors and reviewers. We strongly encourage code deposition in a community repository (e.g. GitHub). See the Nature Research guidelines for submitting code & software for further information.

Data

Policy information about availability of data

All manuscripts must include a data availability statement. This statement should provide the following information, where applicable:
- Accession codes, unique identifiers, or web links for publicly available datasets
- A list of figures that have associated raw data
- A description of any restrictions on data availability

Data supporting the findings of this manuscript are available from the corresponding author upon reasonable request

Field-specific reporting

Please select the one below that is the best fit for your research. If you are not sure, read the appropriate sections before making your selection.

☒ Life sciences ☐ Behavioural & social sciences ☐ Ecological, evolutionary & environmental sciences

For a reference copy of the document with all sections, see nature.com/documents/nr-reporting-summary-flat.pdf

Life sciences study design

All studies must disclose on these points even when the disclosure is negative.

Sample size	Sample sizes were not pre-determined for these analyses. For animal studies, 3 biological replicates was selected as a self-imposed minimum. In all experiments, sample size (and therefore study power) was deemed sufficient if significant differences could be identified.
Data exclusions	Sensors that were sheared during injection were excluded from these analyses: these sensors were clearly not spherical (crescent-moon shaped), and can be easily identified and excluded from the dataset.
Replication	Key experiments reported in the main manuscript were repeated successfully at least three times.
Randomization	For mouse studies, mice were randomly assigned treatment condition and time point. For spheroid studies, only spheroids that had µTAMs incorporated were selected for residual elasticity measurements. Spheroids that did not have any sensors were not studied.
Blinding	Blinding is not relevant to this analysis, as there were no comparative conditions to blind. The only possible blind would be to compare spheroid types; but the resulting spheroid morpohologies are so distinctive that blinding would not provide any advantage.

Reporting for specific materials, systems and methods

We require information from authors about some types of materials, experimental systems and methods used in many studies. Here, indicate whether each material, system or method listed is relevant to your study. If you are not sure if a list item applies to your research, read the appropriate section before selecting a response.

Materials & experimental systems

n/a	Involved in the study	
☒	☐	Antibodies
☐	☒	Eukaryotic cell lines
☒	☐	Palaeontology and archaeology
☐	☒	Animals and other organisms
☒	☐	Human research participants
☒	☐	Clinical data
☒	☐	Dual use research of concern

Methods

n/a	Involved in the study	
☒	☐	ChIP-seq
☒	☐	Flow cytometry
☒	☐	MRI-based neuroimaging

Eukaryotic cell lines

Policy information about cell lines

Cell line source(s)	ATCC: HS-5(CRL-11882); T47D (CRL-11882); MDAMB-231 (HTB-26), 4T1 (CRL-2539)
Authentication	The cell lines used were not authenticated
Mycoplasma contamination	Cell lines were not tested for mycoplasma contamination, but no evidence of contamination was observed
Commonly misidentified lines (See ICLAC register)	None

Animals and other organisms

Policy information about studies involving animals; ARRIVE guidelines recommended for reporting animal research

Laboratory animals	8-12 week old female BALB/c mice (Charles River) were used. Details on animal housing conditions provided in manuscript.
Wild animals	No wild animals were used in this study
Field-collected samples	No field-collected samples were used in this study.

Ethics oversight	Animal Resource Center of McGill University

Note that full information on the approval of the study protocol must also be provided in the manuscript.
